# Exercise improves the quality of slow-wave sleep by increasing slow-wave stability

**DOI:** 10.1038/s41598-021-83817-6

**Published:** 2021-02-24

**Authors:** Insung Park, Javier Díaz, Sumire Matsumoto, Kaito Iwayama, Yoshiharu Nabekura, Hitomi Ogata, Momoko Kayaba, Atsushi Aoyagi, Katsuhiko Yajima, Makoto Satoh, Kumpei Tokuyama, Kaspar E. Vogt

**Affiliations:** 1 International Institute for Integrative Sleep Medicine (WPI-IIIS), University of Tsukuba, 1-1-1 Tennodai, Tsukuba, Ibaraki Japan; 2 Faculty of Budo and Sport Studies, Tenri University, Tenri, Japan; 3 Faculty of Health and Sports Sciences, University of Tsukuba, Tsukuba, Japan; 4 Graduate School of Humanities and Social Sciences, Hiroshima University, Hiroshima, Japan; 5 Department of Somnology, Tokyo Medical University, Tokyo, Japan; 6 Faculty of Pharmaceutical Sciences, Josai University, Saitama, Japan

**Keywords:** Sleep, Slow-wave sleep

## Abstract

Exercise can improve sleep by reducing sleep latency and increasing slow-wave sleep (SWS). Some studies, however, report adverse effects of exercise on sleep architecture, possibly due to a wide variety of experimental conditions used. We examined the effect of exercise on quality of sleep using standardized exercise parameters and novel analytical methods. In a cross-over intervention study we examined the effect of 60 min of vigorous exercise at 60% $\dot{V}{\text{O}}_{2}$max on the metabolic state, assessed by core body temperature and indirect calorimetry, and on sleep quality during subsequent sleep, assessed by self-reported quality of sleep and polysomnography. In a novel approach, envelope analysis was performed to assess SWS stability. Exercise increased energy expenditure throughout the following sleep phase. The subjective assessment of sleep quality was not improved by exercise. Polysomnography revealed a shorter rapid eye movement latency and reduced time spent in SWS. Detailed analysis of the sleep electro-encephalogram showed significantly increased delta power in SWS (N3) together with increased SWS stability in early sleep phases, based on delta wave envelope analysis. Although vigorous exercise does not lead to a subjective improvement in sleep quality, sleep function is improved on the basis of its effect on objective EEG parameters.

## Introduction

Epidemiologic studies indicate that insufficient sleep and/or poor sleep quality are associated with multiple adverse effects on health, such as an increased risk for hypertension, type 2 diabetes, and obesity^1–4^. Insufficient sleep is also associated with anxiety, depression, and an increased risk for other psychiatric disorders^5–7^. Physical exercise is recommended by academic sleep associations as a low-cost, easily administered, and non-pharmacologic intervention for improving sleep^8–11^. A number of studies have demonstrated that a single bout of exercise can decrease sleep onset latency and wake after sleep onset while simultaneously increasing sleep efficiency and slow-wave sleep (SWS)^12–15^. Some studies also report that repeated exercise can induce more salient, chronic effects on the sleep architecture^14,15^. Other studies, however, report few, or even adverse, effects of exercise on the sleep architecture. In healthy young participants, SWS duration was decreased by moderate exercise with an intensity of 35%-45% of maximal oxygen consumption ($\dot{V}{\text{O}}_{2}$max)^16,17^. Another study reported no significant differences in the total sleep time and SWS in healthy young men and women exercising at 45%, 55%, 65%, or 75% of the $\dot{V}{\text{O}}_{2}$max compared to a trial without exercise^18^. Yet another study reported that 12 weeks of exercise training did not alter the duration of SWS and sleep latency in young female participants^19^. Although several investigators have attempted to explain these discrepancies by examining differences in experimental protocols such as the sex, age, and exercise habits of the participants, and in the exercise regimen (type, intensity, duration of exercise, and time of day to exercise), the discrepancies in the effects of exercise on sleep remain to be fully explained.

For more than half a century, since 1968, sleep has been evaluated by applying standardized scoring criteria to electroencephalogram (EEG) and electromyogram recordings established by Rechtschaffen and Kales^20^. We hypothesized that the discrepancies in the effect of exercise on sleep architecture may at least in part originate from the semi-quantitative nature of this sleep-stage scoring. For example, an epoch is scored as sleep stage N3 or SWS when slow-wave EEG (0.5–2 Hz) with an amplitude greater than 75 µV is observed for more than 20% of a 30-s epoch. A further increase in the amplitude or duration of EEG slow waves does not affect sleep scoring, thereby potentially masking meaningful effects.

As a more quantitative approach to determining sleep depth and quality^21^, the delta (δ) power (typically 0.5–4 Hz) of the EEG is evaluated using fast-Fourier transformation^22^. Studies assessing the effects of exercise on EEG δ power have produced mixed results. A number of studies demonstrated that exercise is associated with an increase in δ power during subsequent sleep^23,24^. Young, fit participants also exhibited increased δ power (0–3.9 Hz) after a 30- or 42-km cross-country running race^23^. In addition, a recent study showed that δ power (0.5–4 Hz) was increased by a moderate (40% of $\dot{V}{\text{O}}_{2}$max) bicycle ergometer workout in healthy male participants^24^. In another study, trained athletes who exercised daily at moderate to high intensity were requested to remain sedentary in the laboratory for an entire day, and investigators found no significant differences in the δ power (0.33–3 Hz) between the exercise and sedentary days^25^.

A novel computational method for analyzing EEG waves based on envelope analysis was proposed in 2018^26^. The envelope of a signal in a given frequency band, obtained through its Hilbert transformation, can be viewed as a representation of the instantaneous power in this band. The coefficient of variation of this measure shows how much this power varies over time. The coefficient of variation of the envelope (CVE) thus provides a scale-independent measure of the temporal stability of an oscillation. Low CVE values are found for stable sinusoidal oscillations, intermediate CVE values indicate Gaussian oscillations, and high CVE values are a sign of irregular phasic processes^26^. We used CVE analysis as a novel tool to investigate the effect of exercise on sleep to examine not only the power of the EEG δ waves generated, but also the stability of these waves.

The present study evaluated the effects of a single bout of vigorous exercise in young healthy men on the metabolic state of subsequent sleep and its quality. We wanted to determine, whether exercise improved or decreased sleep quality and whether short exercise bouts can exert lasting effects on the metabolic state.

## Results

### Participant characteristics

**Figure 1:**
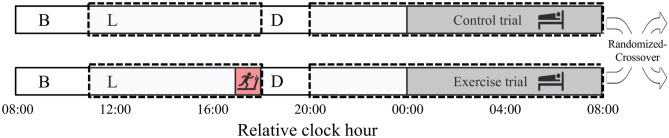
Study protocol. The schedule of the control day (upper bar) and exercise day (bottom bar). For participants whose habitual bedtime is at 00:00, indirect calorimetry begins at 11:00 and ends at 08:00 of the next morning, as shown by the dotted rectangles. Participants exited the metabolic chamber at 19:00 for preparation of the polysomnographic measurement and reentered at 21:00. Gray, red, and white boxes represent sleep (00:00–08:00), exercise (17:00–18:00), and wakefulness (08:00–24:00), respectively. Breakfast, lunch, and dinner are denoted by B, L, and D, respectively.

The participant characteristics were (mean ± SEM): age 23.8 ± 0.7 years, weight 66.6 ± 2.2 kg, body fat 17.6 ± 0.01%, and BMI 22.8 ± 0.6 kg/m^2^. The average $\dot{V}{\text{O}}_{2}$max was 55.27 ± 5.29 ml/kg/min. All participants completed 2 trials, and there were no significant differences in weight, body fat, and BMI among the trials. All participants fulfilled all inclusion/exclusion criteria (Fig. 1).

### Lasting effects on metabolic state

**Figure 2:**
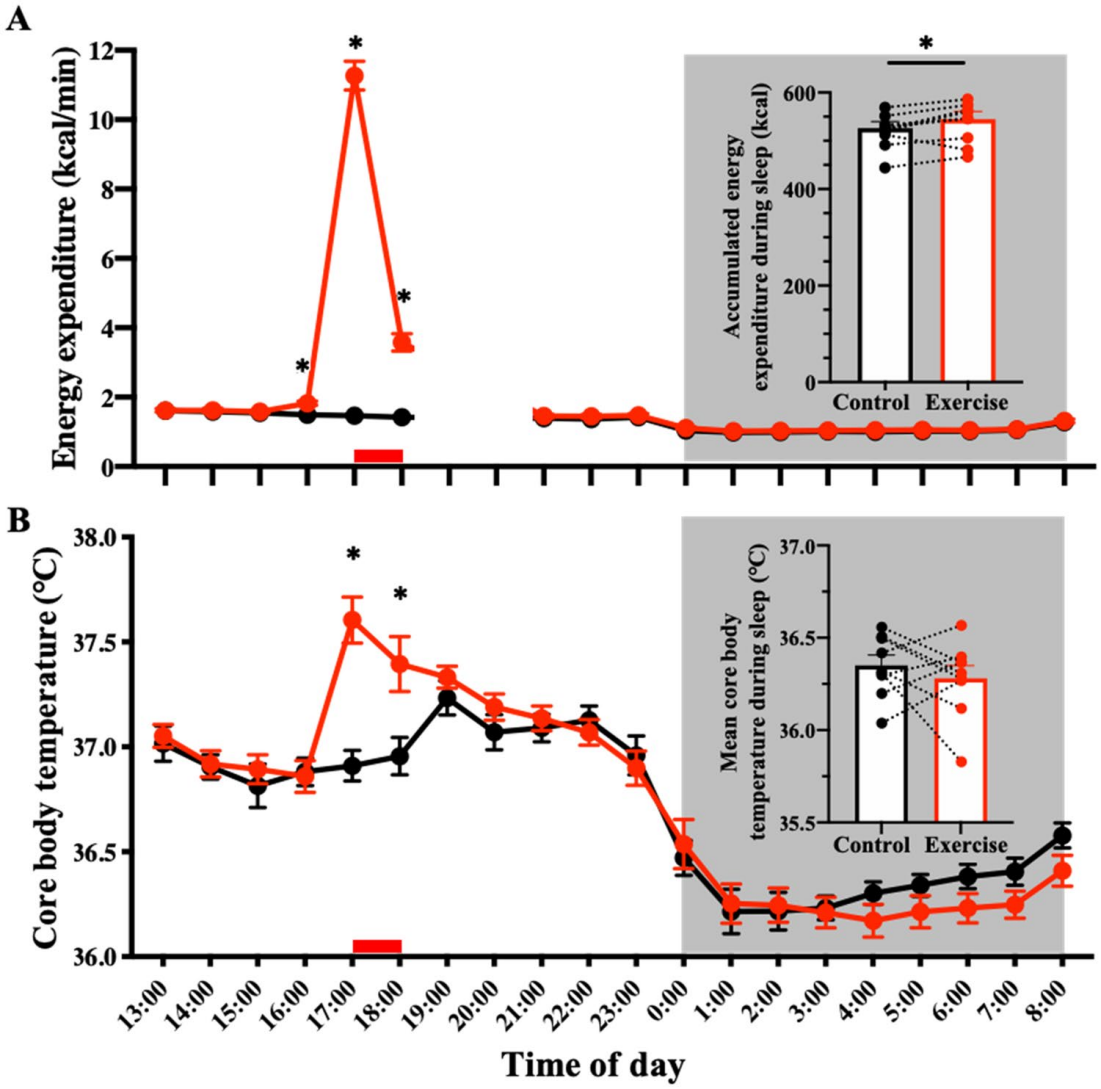
Time-course of energy expenditure and core body temperature. Time-course of energy expenditure (**A**) and core body temperature (**B**) during the entire experiment is shown. Hourly means ± SE are shown for control (filled black circle) and exercise trials (filled red circle), respectively. The red bar at the bottom represents exercise or a sedentary period, and the gray area represents the sleep period. To attach PSG electrodes, participants exited from the metabolic chamber (19:00–21:00). *Represents a statistically significant difference between control and exercise trials by post hoc comparisons using Bonferroni’s correction for multiple comparisons (*p < 0.05).

As expected, energy expenditure increased during the exercise period (control trial: 88 ± 3 kcal/h vs. exercise trial: 676 ± 25 kcal/h, *p* < 0.001; Fig. 2A). As a consequence, oxygen consumption during exercise increased up to 747% (control trial: 0.30 ± 0.01 L/min vs. exercise trial: 2.27 ± 0.08 L/min), HR increased by 238% (control trial: 65 ± 3 beats/min vs. exercise trial: 154 ± 4 beats/min), and core body temperature increased by 0.70 °C (control trial: 36.91 ± 0.07 °C vs. exercise trial: 37.61 ± 0.11 °C) above the sedentary condition. Hourly means of the core body temperature during exercise and 1 h post-exercise were also higher in the exercise trials compared with the control trial. A 2-factor repeated measures ANOVA identified a significant effect of time (*p* < 0.0001) and interaction (p < 0.0001), although the main effects of group were not significant (Fig. 2B).

The mean core body temperature throughout the post-exercise sleep period was not significantly different from that during the control trials (control trials: 36.35 ± 0.06 °C vs. exercise trials: 36.28 ± 0.04 °C, *p* = 0.40). The hourly core body temperature curves during the sleep period differed between the 2 conditions. A 2-factor repeated measures ANOVA showed no effect of exercise (*p* = 0.4119), but a significant effect of time (*p* < 0.0001) and a significant interaction between exercise and time (*p* = 0.007; Fig. 2B). Energy expenditure remained elevated throughout sleep after exercise (control trial: 526 ± 15 kcal/8 h vs. exercise trial: 544 ± 17 kcal/8 h, *p* < 0.05; Fig. 2A). Thus, even several hours after a bout of vigorous exercise, the metabolic state was altered in subsequent sleep.

### Subjective assessment of sleep quality

Subjective sleep quality on the basis of responses to the OSA-MA questionnaire differed for 'Refreshness' and 'Frequent Dreaming or Nightmares' between the exercise and control conditions, with no significant differences in the other parameters ('Sleepiness on Rising', 'Initiation and Maintenance of Sleep', and 'Sleep Length'; Table 1). Thus vigorous exercise did not improve the subjective assessment of the sleep quality.

**Table 1:** Subjective parameters by OSA sleep inventory MA version (mean ± standard error).

Parameters	Control	Exercise	p
	Mean	Mean	
Sleepiness on rising	20.0 ± 1.4	19.5 ± 2.0	0.80
Sleep duration	16.7 ± 1.8	19.1 ± 1.7	0.37
Initiation and maintenance of sleep	16.5 ± 2.5	17.7 ± 2.4	0.46
Refreshness	22.9 ± 1.1	16.8 ± 2.4	0.03*
Frequent dreaming, nightmares	19.6 ± 1.1	23.1 ± 1.4	0.02*

*OSA* The Oguri Shirakawa and Azumi standard rating scale, *MA* middle age and aged version.

*p < 0.05.

### Objective assessment of sleep quality

**Figure 3:**
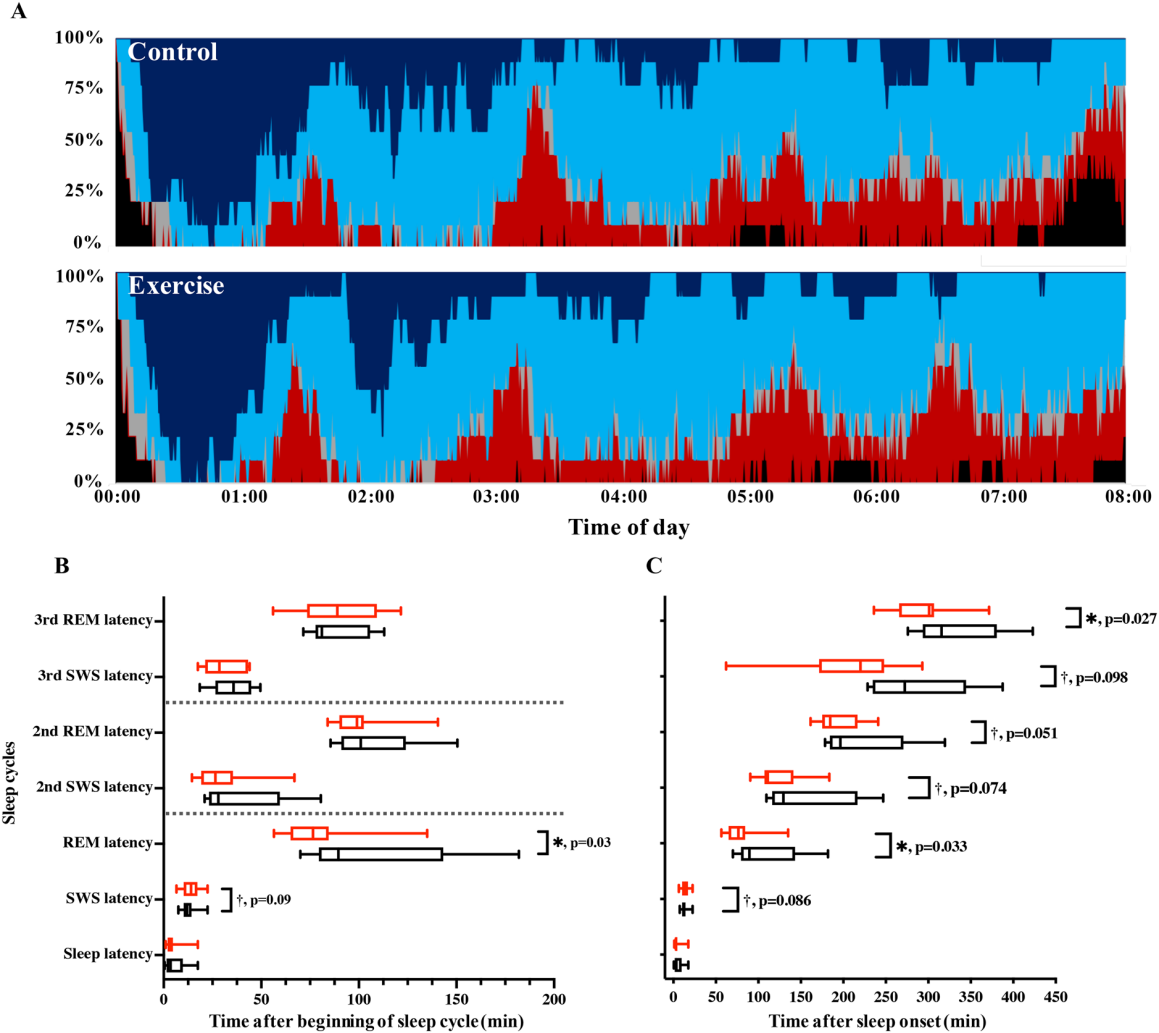
Time-course of sleep architecture and timing of sleep cycles. (**A**) Sleep architecture of the 9 participants for the control (upper panel) and exercise trials (bottom panel). Percentage of participants in stage W (wakefulness; black), stage N1 (gray), stage N2 (light blue), SWS (dark blue), and stage REM (red) changed with the sleep time. B and C: Latencies of SWS and REM sleep evaluated as time after beginning of sleep cycle (**B**) and as time after sleep onset (**C**) are shown. Latency of sleep stage transition in each sleep cycle is shown with black and red box-whisker plots for control and exercise trials, respectively. * and † represent statistically significant differences between the control trial and exercise trial by a paired t-test (*p < 0.05; ^†^*p* < 0.1).

**Figure 4:**
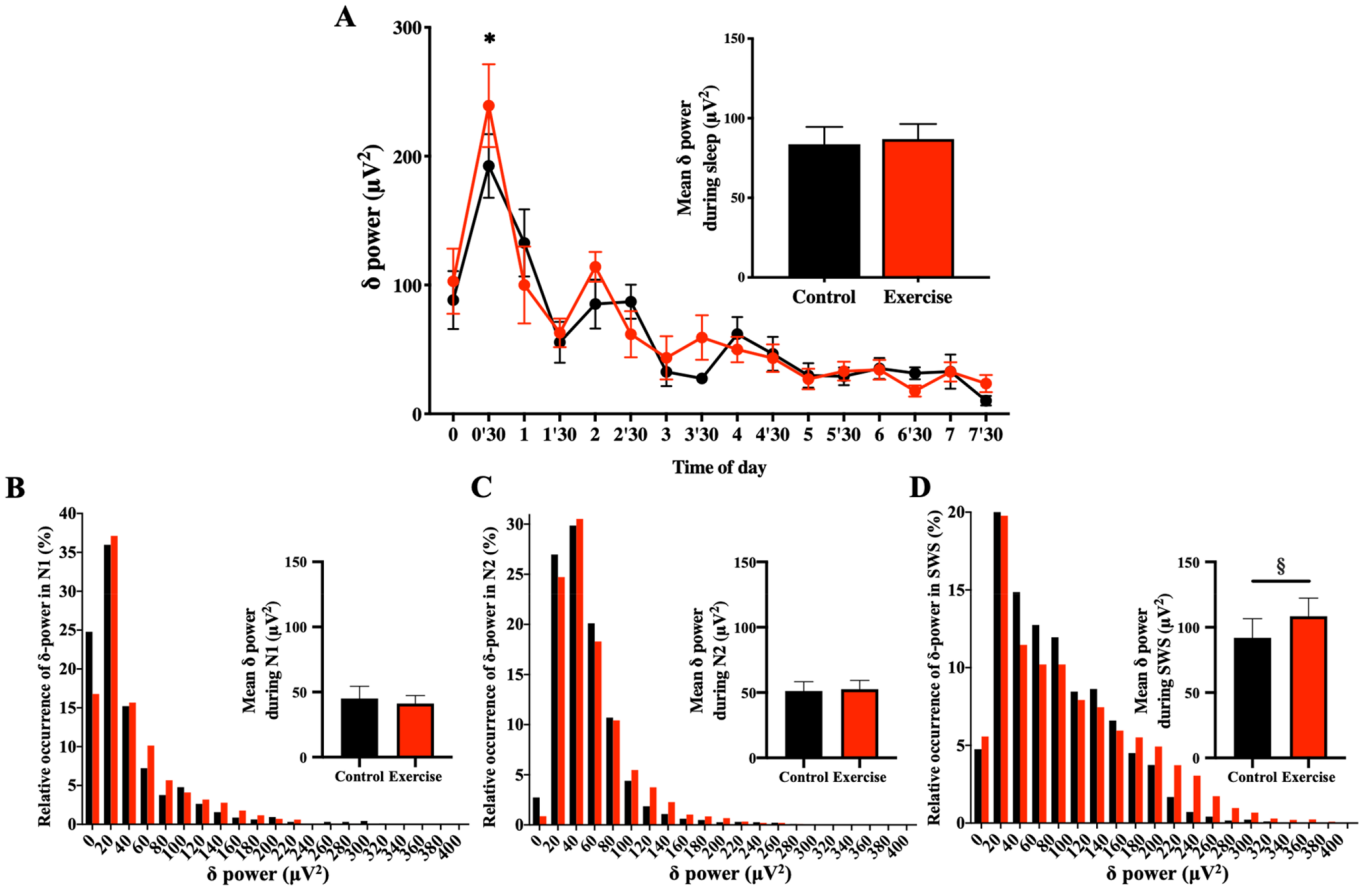
Time-Course of δ-Power of the non-REM Sleep EEG & Relative Occurrence of δ-Power in Each non-REM stage. (**A**) The 30-min means ± SE of δ-power of the 9 participants are shown as a line graph and accumulated δ-power during non-REM is shown as a bar graph. *Represents a statistically significant difference between the control trial and exercise trial by post hoc comparisons using Bonferroni’s correction for multiple comparisons (*p* < 0.05). (**B**–**D**) Relative occurrences of δ-power in N1 (**B**), N2 (**C**), and SWS (**D**) stages are shown. Inserted bar graphs in each panel represent mean δ-power in each non-REM stage. Black plots (filled black circle) and bars (filled black square) represent control trials, and red plots (filled red circle) and bars (filled red square) represent exercise trials. ^§^Represents a statistically significant difference between control trial and exercise trial by a paired t-test (^§^*p* < 0.05).

Basic sleep architecture (i.e., durations of stage 1, stage 2, SWS, REM, and wakefulness after sleep onset) was largely unchanged between the conditions; with the exception of REM, SWS sleep latency, and SWS duration, which were shorter following exercise (Table 2 and Fig. 3). Shortened SWS durations were limited to the first sleep cycle (64.39 ± 5.65 vs. 48.61 ± 3.73 min for control and exercise trials, *p* = 0.019). SWS episode durations in subsequent sleep cycles were not significantly different (20.28 ± 2.42 min vs. 26.17 ± 4.00 min for control and exercise trials during the second cycle, *p* = 0.176; 10.17 ± 2.79 min vs. 10.00 ± 2.72 min for control and exercise trials during the third cycle, *p* = 0.780). At first glance, these results indicate a decrease in slow wave activity. To further evaluate this finding, we investigated the power of the δ oscillations in detail. Overall mean δ power throughout the whole sleep period (control trials: 83.67 ± 10.85 μV^2^ vs. exercise trials: 86.88 ± 9.54 μV^2^, *p* = 0.425) was not significantly different between conditions (Fig. 4A). Interestingly, however, δ power in SWS (N3) was significantly larger in the exercise condition (108.4 ± 13.9 μV^2^) than in the control condition (92.0 ± 14.6 μV^2^; *p* = 0.047). Mean δ power in N1 (45.1 ± 9.3 μV^2^ vs. 41.2 ± 6.2 μV^2^ for control and exercise trials, *p* = 0.645) and N2 (51.2 ± 7.1 μV^2^ vs. 52.6 ± 6.8 μV^2^ for control and exercise trials, *p* = 0.711) was similar between conditions. As a consequence, δ wave energy generated over the shortened SWS period was actually larger in the exercise condition compared with the control condition (Fig. 4B–D).

**Table 2:** Sleep Architecture (mean ± standard error).

Parameters	Control	Exercise	p
	Mean	Mean	
Total bedtime (min)	480.0	480.0	
Total sleep time (min)	449.8 ± 8.1	459.6 ± 5.9	0.14
Wakefulness (min)	21.9 ± 7.1	15.5 ± 4.0	0.36
Sleep latency (min)	5.6 ± 1.9	4.6 ± 1.7	0.43
Sleep efficiency (%)	93.5 ± 1.6	95.5 ± 1.2	0.15
Stage 1 (min)	42.3 ± 5.0	48.2 ± 5.2	0.20
Stage 2 (min)	228.5 ± 10.5	236.6 ± 11.4	0.25
SWS (min)	101.6 ± 7.2	90.8 ± 6.9	0.01**
REM sleep (min)	76.4 ± 4.7	82.6 ± 8.7	0.53
REM sleep latency (min)	107.7 ± 14.2	80.1 ± 7.6	0.03*

*SWS* slow-wave sleep, *REM* rapid eye movement.

*p < 0.05, **p < 0.01.

We also performed a detailed examination of the time course of δ wave power throughout sleep. A 2-factor repeated measures ANOVA revealed a significant effect of time (*p* < 0.0001) and a significant interaction between time and exercise condition (*p* = 0.0198). Post-hoc analysis showed a significant difference in SWS δ wave-power during 00:30–01:00 after sleep in the exercise condition (Fig. 4A). In summary, we found that the generation of δ wave-power was significantly increased in early sleep phases, without an overall increase in EEG δ wave-power throughout sleep in the exercise trials.

**Figure 5:**
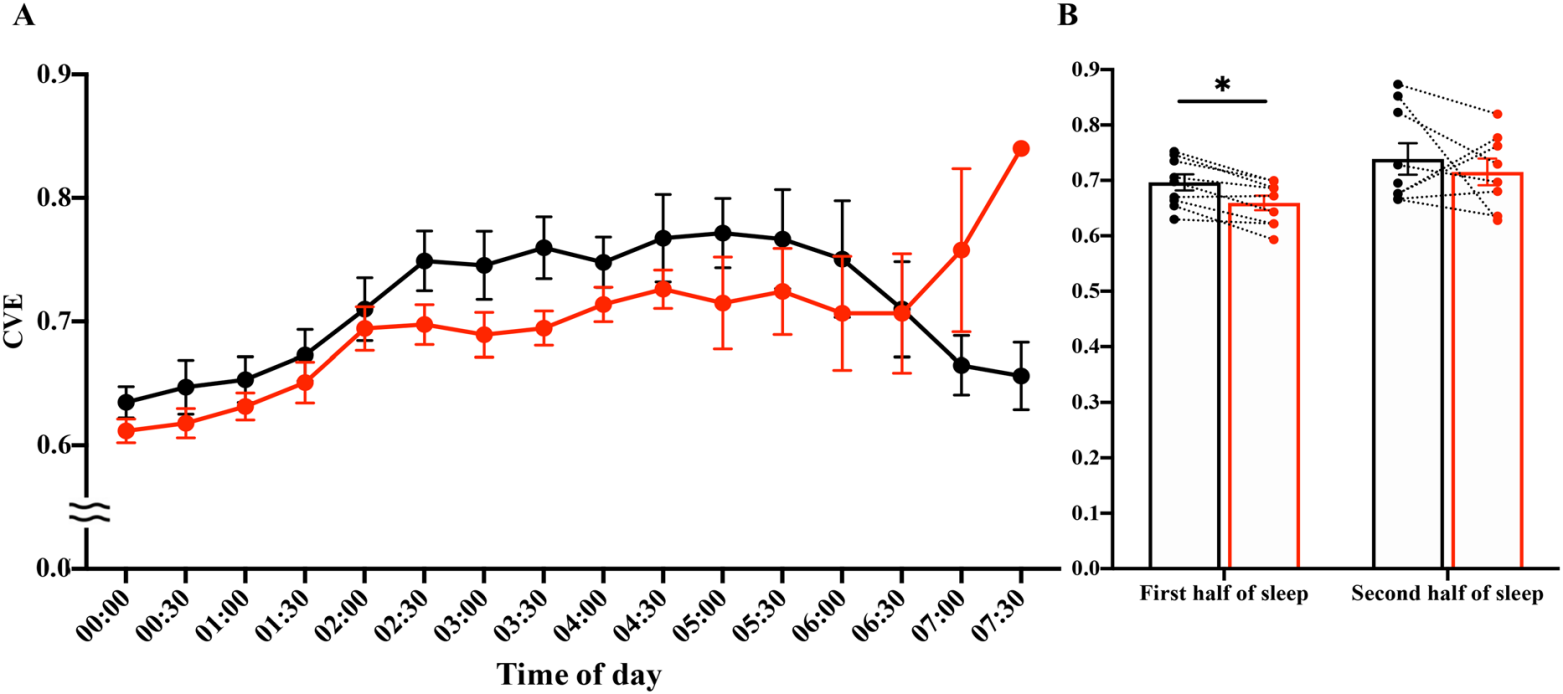
Envelope analysis. (**A**) Time-course of the CVE during the entire sleep. The 30-min means ± SE of the CVE are shown for the control trial (filled black circle) and exercise trial (filled red circle). (**B**) Mean CVE during the first half and second half of sleep are shown. Mean CVE is shown for the control trial (open black square) and exercise trial (open red square). Dotted lines connect the same participants. *Represents a significant difference between the control trial and exercise trial by a paired t-test (*p < 0.05). Note that the CVE values did not differ significantly between control and exercise in the last hour of sleep. CVE values were most likely affected by the very low δ power values during this time.

We subsequently examined the stability of the EEG δ waves using CVE analysis. Low CVE values indicate stable, rhythmic, δ wave oscillations, whereas high CVE values indicate short phasic events in the δ frequency range. In an animal model it has been shown that δ-band CVE converges towards 1 as its minimal possible value (see discussion for details). Here we show that in humans this limit holds and δ-band CVE diminishes with increasing sleep depth (CVE of N1 > CVE of N2 > CVE of SWS; Table 3). Our detailed analysis of SWS revealed a significant effect of time (*p* < 0.0001) and a significant interaction of time and exercise condition (*p* = 0.0265; Fig. 5A). Post-hoc comparisons showed significant differences in multiple comparisons. Specifically, exercise trials were associated with lower CVE values than control trials in the first half of sleep (1.50 ± 0.03 vs. 1.44 ± 0.03 for control and exercise trials, *p* = 0.0051; Fig. 5B). This finding reinforces the notion of increased density and stability of δ wave oscillations in early sleep phases after exercise.

**Table 3:** CVE Values in Each non-REM stage (mean ± standard error).

Non-REM stage	CVE	
	Mean ± sem	Post-hoc comparisons
N1	1.81 ± 0.03	N1 > N2, *p* = 0.586
N2	1.69 ± 0.05	N2 > SWS, *p* = 0.026*
SWS	1.39 ± 0.08	SWS < N1, *p* < 0.001**

*CVE* coefficient of variation of the envelope.

*p < 0.05, **p < 0.01.

## Discussion

The present study investigated the acute effects of a single bout of high-intensity exercise on the subsequent sleep phase, as assessed by observation of the metabolic state, responses to a sleep questionnaire, sleep-stage scoring, EEG spectral analysis, and envelope (CVE) analysis of the EEG δ wave band. The parameters of the single 1-h bout of vigorous exercise chosen here were comparable with those of exercises used in studies registering positive effects of exercise on sleep^12,13,15^ and represent a realistic exercise regimen for healthy adults.

One potential limitation of the study should be considered in the interpretation of the findings. Although not mentioned by the participants, stress due to the unfamiliar sleeping conditions may have affected sleep quality. It should be noted, however, that participants underwent an adaptation day before the experiment and the high sleep efficiency observed in both trials excludes disturbed sleep under the experimental conditions. Moreover, first-night effects would be expected to affect both trial conditions equally owing to the crossover design. To generalize the effects of exercise on sleep, future studies utilizing a different experimental design are warranted, including experiments with a larger sample size. Protocols using regular, chronic exercise with participants of different fitness levels should also be performed.

In the present study, 1 h of vigorous exercise in the evening in untrained volunteers had a moderate, but statistically significant effect on the metabolic state throughout the subsequent sleep phase, detected as excess post-exercise oxygen consumption. Other studies, however, showed that a single bout of low- or high-intensity exercise before lunch did not affect energy expenditure during subsequent sleep^27,28^. Excess post-exercise oxygen consumption can be interpreted as restoring an oxygen deficit incurred during exercise and more complex mechanisms, including factors that directly (e.g., availability of metabolites such as ADP, ATP, inorganic phosphate, and creatine phosphate) or indirectly (e.g., release of catecholamines, thyroxine, glucocorticoids, fatty acids, calcium ions, and temperature [Q_10_ effect]) affect mitochondrial O_2_ consumption^29^. Interestingly, the increase in energy expenditure in sleep after exercise was not accompanied by an increase in the core body temperature, suggesting an important difference in heat dissipation, which was also observed in previous studies^24^.

Post-exercise sleep was judged subjectively worse compared to sleep following non-exercise conditions. We hypothesize that mechanisms underlying the post-exercise oxygen deficit and excess oxygen consumption indicate that subjects are under stress and this might explain this lower subjective assessment regarding the 'Refreshness' category in the exercise condition. Another potential reason for subjectively worse sleep after vigorous exercise is muscle soreness as the participants were not accustomed to vigorous exercise. Indeed, in a previous study of moderate (as opposed to vigorous) exercise (~ 45% $\dot{V}{\text{O}}_{2}$max) in young healthy males, participants reported increased subjective sleep quality, particularly ‘Initiation and maintenance of sleep’^30^. Recommendations for exercise for non-pharmacologic improvement of subjective sleep quality may benefit from suggestions to participate in moderate exercise, at least initially.

While vigorous exercise may be judged as negatively affecting subjective sleep quality by participants, we found that objective measures of sleep quality indicate a more complex picture suggesting an opposite, beneficial effect. Sleep staging according to American Academy of Sleep Medicine criteria revealed little difference between the exercise and control conditions, consistent with previous studies^16–19^. Sleep staging is inherently semi-quantitative, e.g., when the criteria for the SWS stage are fulfilled, further increases in sleep depth cannot be resolved. Notably, stage N4, which might allow for more fine-grained classification of sleep depth, was recently abolished. We observed shortening of the first N3 episode, while several studies investigating the effect of exercise on sleep observed little effect on the total duration of SWS^16–19,31^. The participants in our study were not regularly exercising at the level used in this study, which might explain some differences between our findings and those of previous studies, but a recent meta-analysis indicated that fitness level does not modulate the effect of exercise on SWS^13^.

REM latency is chronically shortened in some pathologic conditions, including depression^32^ and attention-deficit/hyperactivity disorder^33^, but these are unlikely causes in the present study, which included young healthy participants. In fact, physical exercise is a known beneficial intervention for depression^34^. The shortened first REM sleep latency we observed in this study can be interpreted as a forward shift of sleep processes following exercise. Latencies of SWS and REM sleep evaluated as time after the beginning of the sleep cycle did not differ significantly during the second and third sleep stages (Fig. 3). The latencies were shortened, however, when evaluated as time after sleep onset, i.e., indicating a shift forward. A potential caveat of this type of analysis is the necessarily semi-quantitative nature of the scoring system, which may obscure more subtle differences. As a more quantitative measure, energy in the EEG δ power is viewed as the most reliable indicator of sleep-need buildup and resolution^35^. Accordingly, the increased δ wave energy production in the first SWS period observed here indicates a more rapid reduction of sleep need in the early sleep phases after exercise, reinforcing the notion of more efficient early sleep processes. Thus, exercise could help achieve efficient sleep earlier by more effectively reducing the sleep need during the first SWS episode. A recent study showed that exercise performed in the evening delays the nocturnal melatonin rise, indicating an effect on the central clock^36^. Our finding of an advance in the sleep cycle after exercise shows that this mechanism is not responsible for our results.

The lack of an increase in overall δ wave-power throughout the entire sleep period shows that the overall sleep need was not increased by 1 h of vigorous physical exercise. This finding is in contrast to the previous report that sleep after high-intensity exercise (50–70% $\dot{V}{\text{O}}_{2}$max) increased sleep need as defined by enhanced SWS duration^31^, but is consistent with findings from a study reporting no effect of high-intensity exercise (65% and 75% $\dot{V}{\text{O}}_{2}$max) on sleep need^18^.

Complementary to measuring spectral power, envelope analysis, operating in the time domain, allows an even more detailed analysis of EEG activity, providing information about the morphology of slow waves. According to a recent model based on rat EEG recordings, δ waves originate from the superposition of transient events whose density controls the phasic or continuous appearance of the resulting wave as well as its amplitude^26^. A few transients (i.e. isolated slow waves) over the EEG background are reported by CVE $\gg$ 1, while epochs showing dense slow waves are characterized by CVE approaching 1 (for simplicity, CVE = 1 represents a theoretical constant related to Gaussian waves, i.e. the limit for a random superposition of high-density transient events)^26^. In this study we show that in humans NREM stages are arranged on the CVE scale as 1 < N3 < N2 < N1 (Table 3), hence CVE can be directly interpreted as sleep depth. These morphologic variations in δ waves can be followed quantitatively using envelope analysis, which provides the investigator with a novel tool for assessing the effect of manipulations on sleep that may otherwise elude detection. As a general observation, deep SWS is accompanied by lower CVE values compared with shallow non-REM sleep. The lower CVE values in SWS that we observed here together with the higher δ wave energy in the first SWS period reinforce the notion that the processes generating slow waves are more efficient after exercise compared with control conditions. To our knowledge this is the first report of exercise exerting such an effect. Further investigation into the mechanisms and consequences of this increased δ wave stability are necessary.

Patients who need or wish to perform vigorous exercise during the day may judge their subsequent sleep as inferior compared to rest. Our results indicate that objective parameters contradict this subjective assessment and may serve to reassure individuals, such as athletes who need or wish to perform at high $\dot{V}{\text{O}}_{2}$max loads, that, if anything, sleep is improved by their physical exercise.

## Methods

### Participants

Nine healthy young men participated in the study. All participants satisfied the inclusion criteria, as follows: 20–30 years of age, body mass index of 18.0–29.9 (kg/m^2^), a regular sleep/wake pattern, and regular exercise no more than twice a week. Exclusion criteria for the study participants was determined following previous studies^22,41^; self-reported sleep problems (Pittsburgh Sleep Quality Index score > 5); shiftwork or transmeridian travel within 1 month before the study; smoking; excessive alcohol intake (> 30 g alcohol/day); ongoing medication for cardiovascular disease, diabetes, hypercholesterolemia, hyperglycemia, or hyperlipidemia; and the use of medications affecting sleep or metabolism. Based on sample size calculation, our 9 participants allow us to observe a significant difference with a paired t-test with 75% power and 5% alpha level. Power analysis was conducted by using G-Power 3.1.9 software. This study was conducted according to the guidelines of the Declaration of Helsinki and all procedures involving human participants were approved by the Ethics Committee of the University of Tsukuba. The study protocol was approved by the University of Tsukuba (approval number: tai-28-52) and registered with Clinical Trials UMIN (ID numbers: UMIN000040428, 31/05/2020). All participants provided written informed consent before study commencement.

### Procedures

The present study was a randomized-crossover intervention study. The 2 trials were separated by a washout period of 1 week. All participants performed a graded exercise test comprising submaximal and maximal tests using a treadmill (ORK-7000, Ohtake-Root Kogyo Co., Ltd, Iwate, Japan)^37^ to determine a workload corresponding to 60% of each individual’s $\dot{V}{\text{O}}_{2}$max. The test was performed within a month before the first experimental trial. Additionally, the experiment was preceded by an adaptation night in the whole-room metabolic chamber, during which the sensors and electrodes of the polysomnographic recording system were attached to the participants. For 5 days prior to the experiment, participants maintained a constant 8-h sleep/16-h wake schedule following their habitual bed and awake time. The participants refrained from ingesting beverages containing caffeine and alcohol, and from performing high-intensity physical activity. Compliance with the instructions was confirmed by sleep diaries and wrist actigraphy (ActiGraph, Ambulatory Monitoring, NY). One day before the experiment and during the experiment day, the participants consumed specified meals at the designated time for breakfast (1 h after waking), lunch (4 h after waking), and dinner (5 h before bedtime).

On the experiment day, the participants arrived at the laboratory, ate lunch, swallowed a core body temperature sensor, and entered the metabolic chamber. The participants performed physical exercise at 60% of the $\dot{V}{\text{O}}_{2}$max for 60 min beginning at 6 h before bedtime using a treadmill (T1201, Johnson Health Tech Japan, Tokyo, Japan) or remained seated. After the exercise period, the participants were allowed to leave the chamber for 90 min to wipe away sweat and eat dinner. After fitting the participants with the electrodes for polysomnography, they entered the metabolic chamber and remained sedentary. The participants went to bed at their usual bedtime (23:30 ~ 24:30) and slept for 8 h. Energy metabolism was measured for 16 h (from lunch to the next morning; Fig. 1).

The specified meals provided were based on energy requirements estimated from the basal metabolic rate^38^ with a physical activity level of 1.3 on the day prior to the experiment and control trials. The physical activity level of the exercise trial was assumed to be 1.64 to maintain a stable energy balance^39^. The macronutrient composition of the meals was 15% protein, 25% fat, and 60% carbohydrates.

### Measures

#### Indirect calorimetry

The airtight metabolic chamber measured 2.00 × 3.45 × 2.10 m (FHC-15S, Fuji Medical Science Co., Ltd., Chiba, Japan), and air in the chamber was pumped out at a rate of 80 L/min. The temperature and relative humidity of the incoming fresh air were controlled at 25 °C and 55%, respectively. The chamber was furnished with an adjustable hospital bed, desk, chair, and toilet. Concentrations of oxygen (O_2_) and carbon dioxide (CO_2_) in the outgoing air were measured with high precision by online process mass spectrometry (VG Prima δB; Thermo Electron Co., Winsford, UK). The precision of the mass spectrometry, defined as the standard deviation for continuous measurement of the calibrated gas mixture (O_2_, 15%; CO_2_, 5%), was 0.0016% for O_2_ and 0.0011% for CO_2_. Every minute, O_2_ consumption ($\dot{V}{\text{O}}_{2}$) and CO_2_ production ($\dot{V}{\text{CO}}_{2}$) rates were calculated using an algorithm for improved transient response^40^. Energy expenditure was calculated from $\dot{V}{\text{O}}_{2}$, $\dot{V}{\text{CO}}_{2}$, and urinary nitrogen excretion (N), as described previously^22,39,41^.

#### Core body temperature

Core body temperature was continuously monitored using an ingestible temperature sensor that wirelessly transmitted the core body temperature to a recorder (CorTemp, HQ Inc, FL, USA). The sensor was accurate to ± 0.1 °C, and was calibrated by immersion in water at a known reference temperature before use and swallowed 4 h before experiment^42^.

#### Self-reported quality of sleep

The Pittsburgh Sleep Quality Index was used to assess sleep quality and sleep disorders in the month prior to the experimental procedures. We assessed 7 components: subjective sleep quality, sleep latency, sleep duration, sleep efficiency, sleep disturbances, use of sleep medication, and daytime dysfunction. The scores of the 7 components were summed to produce a total score (range = 0—21). This index was used only in the preselection stage^43^. The Oguri-Shirakawa-Azumi sleep inventory MA version (OSA-MA) was used to assess subjective sleep quality after waking in the morning^44^. This questionnaire comprises 16 items with 5 factors (‘Sleepiness on rising’, ‘Initiation and maintenance of sleep’, ‘Frequent dreaming’, ‘Refreshness’, and ‘Sleep length’).

#### Polysomnography

The recording system (Alice 5, Respironics Inc, Japan) comprised 6 electroencephalography locations (C3-A2, C4-A1, O2-A1, O1-A2, F3-A2, and F4-A1), submental electromyography, and a bilateral electrooculogram. Sleep parameters were categorized at 30-s intervals as wakefulness and stages N1, N2, SWS, and rapid eye movement (REM) sleep according to the standard criteria of the American Academy of Sleep Medicine^45^. In addition, total sleep time, sleep onset latency, REM sleep latency, and sleep efficiency were evaluated.

#### Data analysis: spectral analysis of the electroencephalogram

The C3-A2 EEG recording was analyzed using discrete fast-Fourier transformation techniques as previously described^22^. Fast-Fourier transformation was conducted on an EEG record length of 5 s to obtain a frequency resolution of 0.2 Hz. Each 5-s EEG segment was first windowed with a Hanning tapering window prior to computing the power spectra. The spectral distribution was categorized into the following frequency bands: delta (δ: 0.75–4.00 Hz), theta (θ: 4.10–8.00 Hz), alpha (α: 8.10–12.00 Hz), sigma (σ: 12.10–14.00 Hz), and beta (β: 14.10–30.00 Hz)^22^. The power content of the δ band for each 30-s epoch of sleep was determined as the mean of the δ power measured in six consecutive 5-s segments of the EEG (expressed as μV^2^).

#### Envelope analysis

The CVE for the δ band was calculated for EEG recordings (C3-A2) at 30-s intervals. To minimize aliasing effects, the epochs had 50% overlap (i.e., epoch length = 60 s). First, every epoch was digitally bandpass-filtered (0.5–4 Hz) with a fourth-order IIR implementation of a Butterworth filter using the 'signal' package for the R language (http://r-forge.r-project.org/projects/signal/). The envelope of the filtered EEG (filt_EEG_envelope) was obtained using its Hilbert transform (Ht) according to the standard relation:

$${\text{Filt}}\_{\text{EEG}}\_{\text{envelope }} = {\text{ sqrt }}\left( {{\text{filt}}\_{\text{EEG}}^{{2}} + {\text{ Ht}}\left( {{\text{filt}}\_{\text{EEG}}} \right)^{{2}} } \right),$$

where sqrt corresponds to the square root. Both the filter and envelope calculations usually produce artifacts at the border of each epoch. To avoid this problem, the samples of each epochs were collected with a 10% excess (i.e., totaling 66 s, 3 s per side). Once the envelope was obtained, this time excess was excised. The mean and standard deviation (SD) of the obtained envelope were calculated and a normalized version of the coefficient of variation (CVE) was obtained sd/(mean*0.523); with 0.523 being the value for Gaussian waves. As a consequence, CVE values larger than 1 result from processes more phasic than Gaussian waves, while values below 1 indicate more sinusoidal processes. For each epoch, the coefficient of variation (i.e. SD/mean) of the corresponding envelope was stored as a relevant feature^26^.

### Statistical analysis

The results are expressed as the mean ± standard error of the mean (SEM). Paired Student's t tests were used to compare the total amount of δ power during the whole sleep period, each sleep stage latency, the OSA-MA parameters, and the sleep parameters between the mean value of trials. The effects of exercise on the time course of δ power, CVE, core body temperature, and energy expenditure were assessed by 2-way repeated-measures analysis of variance (ANOVA) and Bonferroni’s correction for multiple comparisons. 1-way ANOVA and Bonferroni’s correction for multiple comparisons were used to compare the CVE in each non-REM stage. Data analysis was conducted using Prism 8 (GraphPad Software, San Diego, CA), or R (https://www.r-project.org/), and differences were considered significant when the error probability was less than 0.05.
